# Work ability and cognitive impairments in young adult cancer patients: associated factors and changes over time—results from the AYA-Leipzig study

**DOI:** 10.1007/s11764-021-01071-1

**Published:** 2021-06-12

**Authors:** Hannah Brock, Michael Friedrich, Annekathrin Sender, Diana Richter, Kristina Geue, Anja Mehnert-Theuerkauf, Katja Leuteritz

**Affiliations:** Department of Medical Psychology and Medical Sociology, University of Leipzig, Philipp-Rosenthal-Str. 55, 04103 Leipzig, Germany

**Keywords:** Work ability, Cognitive impairments, AYA, Cancer, Young adult, Longitudinal

## Abstract

**Purpose:**

Although young adults represent a significant portion of the working population, little is known about the work ability and cognitive impairments in adolescent and young adult (AYA) cancer patients (including the long-term course) following cancer treatment.

**Methods:**

As part of the AYA-Leipzig study, we surveyed AYA cancer survivors (aged 18–39 years at diagnosis; time since diagnosis ≤ 4 years, including all cancer diagnoses) at two time points (t2 = 12 months after t1). Work ability (Work Ability Index, WAI-r) and cognitive impairments (Copenhagen Psychosocial Questionnaire, COPSOQ) were compared at both time points. We analysed predictors for work ability at, on average, 2 years post cancer diagnosis (t2) by using multivariate regression analyses.

**Results:**

A total of 502 patients (74.7% women) participated in both measurements. Mean work ability increased significantly from t1 (6.0; SD = 2.3) to t2 (6.8; SD = 2.2) (t = −9.3; p < 0.001). As many as 380 (76%) AYA cancer survivors reported reduced work ability at t1; 1 year later, this still applied to 287 (57%) of them. Decreased work ability (t2) was associated with more cognitive impairment, higher effort coping with the disease, comorbidities, sick leave > 6 months (since diagnosis), and having children (adj *R*^2^ = .48). Cognitive impairments occurred in approximately every fifth patient at both surveys.

**Conclusions:**

Achieving maximum work ability is a major challenge for AYAs. Our results show the need for multimodal cancer survivorship and rehabilitation programmes with a special focus on improving cognitive and psychosocial functioning.

**Implications for Cancer Survivors:**

AYAs with cancer should receive targeted occupational counselling early in the course of the disease.

**Supplementary Information:**

The online version contains supplementary material available at 10.1007/s11764-021-01071-1.

## Background

The National Cancer Institute (NCI) refers to young adult cancer patients between the ages of 15 and 39 as adolescents and young adults (AYAs) [1]. In Germany, approximately 3% of new cancer cases affect this age group [2]. AYAs with cancer have been considered a distinct group in oncological research for a number of years, as they differ from paediatric and elderly cancer patients in biological, medical, and psychosocial terms. In addition to the burden that cancer brings in the acute and long term, AYAs are also confronted with age-related challenges, as they generally are in a sensitive developmental phase in their professional careers. They represent a significant portion of the working population and have many years of work ahead [3]. Furthermore, in view of the high survival rates of AYAs, approximately 80% (for all forms of cancer) [4], successful professional reintegration is important from both an individual and a societal perspective [5, 6]. In addition to providing financial security, work fulfils important functions, such as conveying an identity and creating a social network [7]. Not only for this reason, cancer patients’ return to work after successful treatment is seen as a symbol of recovery and is associated with normality [5, 8]. However, given that AYAs are only at the beginning of their professional careers due to their age, the possible consequences of cancer often have more of a long-term impact on them than on older patients.

The professional situation of working adults has been the subject of a large number of studies, yet that of AYAs has hardly been investigated. Approximately 87% of the AYAs return to education or employment within 1 year of oncological rehabilitation [9], but some studies have suggested that the employment rates of AYA cancer survivors are lower than in the general population [7, 9, 10]. Nevertheless, the employment rate must be distinguished from work ability (WA), with the latter being understood as an individual’s ability to cope with professional demands [11]. Only two studies on AYAs [9, 12] have explicitly addressed the construct of WA and used validated instruments or quantitative methods. Approximately 36–38% of AYAs report limited WA after completing their cancer treatment [9, 12]. Another study with older cancer patients has indicated that WA improves with time after diagnosis [13]. Dahl et al. [12] found that comorbidities, cardiovascular disease, low educational attainment, subjective poor health, and increased depression are associated with decreased WA among AYAs as much as 16 years after diagnosis.

In this study, cancer-related cognitive impairments (CRCI) are considered separately as another potential factor that can influence WA. While CRCI has been well researched in paediatric and elderly cancer patients, there has been comparatively little focus on CRCI in AYAs. This is surprising in the light of the AYA-HOPE study, which indicated that up to 53% of the AYAs face treatment-related cognitive impairments even years after diagnosis [14]. In addition, AYA cancer survivors show higher rates of cognitive dysfunction compared to the general population [15]. Thus, cognitive impairment is a common and persistent problem that may also disrupt the AYAs’ work ability [16]. We placed a special focus on CRCI because they can affect successful employment in the workforce and represent one of the greatest barriers to occupational reintegration for AYA cancer survivors [9]. Furthermore, poor cognitive functioning is associated with higher rates of unemployment [7]. However, the impact of cognitive impairments in AYAs on work-related outcomes is not well understood.

Indeed, the limited evidence regarding WA and CRCI in AYA cancer survivors does not allow for specific hypotheses and conclusions. Furthermore, the few existing AYA studies generally focused on the immediate post-diagnosis or -treatment period. There is a great need for empirical research with longitudinal designs in large and representative AYA samples, as they can map the long-term changes of WA and CRCI during this vulnerable period of the development of this understudied population. Due to their tender age, AYAs are at the start or in the middle of their professional careers, which is why the occupational consequences of cancer have greater impact on AYAs than on older patients. Therefore, longitudinal research results are a prerequisite for establishing age-appropriate support services, including screening and preventive tools in a longer-term perspective.

For this reason, the aim of this study was to systematically investigate the extent of WA and CRCI in AYA cancer survivors. We also aimed to measure whether WA and CRCI change over time and analysed sociodemographic, medical, and psychosocial variables that may be associated with WA in the long term.

## Method

### Study design

This study constituted part of the AYA-Leipzig study (AYA-LE), a German prospective longitudinal study that examined different aspects of the life situation, such as quality of life, mental health, and psychosocial needs of AYA cancer survivors at two measurement points [17]. The first time point (t1) extended from May 2014 to December 2015. The follow-up survey (t2) was done, on average, 12 months later and completed in December 2016. The inclusion criteria for participation were as follows: (i) cancer diagnosis (first manifestation, all malignant tumour identities ICD-10 C00-C97), (ii) age at cancer diagnosis 18–39 years, and (iii) a cancer diagnosis within the last 4 years. The study received the approval of the ethics committee of the medical faculty of the University of Leipzig (Ref. No. 372-13-16122013).

### Recruitment

The recruitment of AYAs with cancer was conducted nationwide in collaboration with 16 acute oncological clinics, four rehabilitation clinics, and two tumour registries. All collaborating clinics were provided with full insight into the ethics application and approval for the study. In the acute and rehabilitation clinics, potential candidates who met the inclusion criteria were informed about the study by their treating physicians, nursing staff, or clinical psychologists and invited to participate. Furthermore, interested patients were given the opportunity to apply to participate via social media (project website and Facebook). After providing written informed consent to participate, the patients received either a link to a standardised questionnaire online at LimeSurvey or, if desired, were mailed a printed version of the questionnaire. One year after the t1 survey, the participants were again invited to complete the online version or the printed version of the questionnaire for the t2 survey.

All participants received an expense allowance of 10 EUR at both measurement dates for completing the questionnaire. A detailed description of the study’s recruitment procedure has previously been published by Leuteritz et al. [18].

### Data collection

The data were collected using standardised validated measuring instruments. In addition, various sociodemographic and medical variables were recorded with self-developed questions.

### Work ability

WA was measured with one item from the Work Ability Index (WAI) [19]. The WAI is an established survey instrument used in clinical and workplace health promotion and research, and there is already a German translation [20]. It shows the extent to which an employee is able to perform his or her work in view of their personal circumstances and working conditions [21]. The original questionnaire consists of seven items, and it has been validated in large Finnish longitudinal studies, has a high predictive power for the future course of WA and is considered reliable (Cronbachs α = .83) [21–23]. In our study, we used WAI item 1 for economic reasons (‘current WA compared with the life-time best’, with a possible score of 0 (‘completely unable to work’) to 10 (‘WA at its best’) to validate subjective self-assessment of WA. The WAI item 1 correlates highly with the overall index of the WAI scale and has been used in a number of studies as an economic alternative to the WAI scale to assess WA [24, 25]. The WAI item 1 is also considered to have the highest statistical selectivity [26]. A cut-off was used (WAI <8 = low WA and ≥8 high WA) for WAI item 1 to classify WA [12, 27]. WAI item 1 (measured at t2) also served as a dependent variable in the analysis.

We also used the adapted WAI item 2 (‘My job challenges me…’) to determine whether demands at work cause (1) ‘more physical than psychological’, (2) ‘more psychological than physical’, or (3) ‘equally physical and psychological’ strain.

### Cognitive impairments

The subjective extent of CRCI was assessed with the four-item subscale of cognitive impairments from the Copenhagen Psychosocial Questionnaire (COPSOQ) [28]. This standardised self-assessment tool is a well-established and standardised instrument used to record mental stress at work. A systematic literature review on work-related questionnaires in cancer patients revealed that the COPSOQ has been used in several studies in the context of rehabilitation research [29]. We used the four-item cognitive impairments subscale instead of the entire COPSOQ to measure cognitive impairments, mainly for economic reasons. In the German version, the subscale of cognitive impairments shows a Cronbachs α of between α = .85 and .87 [30]. Respondents could answer the following question on a five-level Likert scale (1 = always to 5 = never/hardly ever): ‘How often during the past 4 weeks have you (1) had problems concentrating, (2) had difficulty with making decisions, (3) had difficulty with remembering, and (4) found it difficult to think clearly?’ As recommended by the authors, the average overall score across all four subcategories of the cognitive impairments subscale was used for evaluation. This was additionally standardised to a value range from 0 to 100 (020 = never/hardly never, 21–40 = seldom, 41–60 = sometimes, 61–80=often, 81–100 = always), whereby a larger value indicates a higher load to ensure comparability with other studies [30].

### Sociodemographic and medical variables

The sociodemographic variables examined in this study were (among others) sex, level of education, children, and monthly net income per household. The medical variables surveyed were comorbidities (‘Do you currently suffer from any other serious physical or mental illnesses?’), chemotherapy, and time of sick leave. The variables were collected by self-report.

### Perceived adjustment to chronic illness

Effort coping with the disease was assessed with the single-item self-assessment Perceived Adjustment to Chronic Illness Scale (PACIS) [31]. Patients were asked to answer the question ‘How much effort does it cost you to cope with your illness?’ and had to indicate a value between 0 (‘none’) and 100 (‘a great deal’). The PACIS item is considered to be a global indicator of disease management, is suitable for use in clinical trials, and has already been validated in many cancer populations [31].

### Statistical analyses

We used IBM SPSS Statistics 25 for statistical data analyses. The means and standard deviations of WA and CRCI (t1 and t2) were calculated for the total sample and for specific subgroups (e.g. sex, age, tumour). To analyse frequency differences, we used chi-square tests and the corresponding phi coefficient to assess effect sizes: φ ≥ 0.1 (small), φ ≥ 0.3 (medium), and φ≥0.5 (high). The mean group differences between subgroups (e.g. sex, age, tumour) were tested at t2, using one-way analysis of variance (ANOVA). The partial eta squared (η^2^) was used to classify the effect size (small: η^2^ ≤ 0.06, medium: η^2^ ≤ 0.14, high: η^2^ > 0.14) [32]. Differences between baseline and follow-up WA and CRCI were tested using Student’s t-test or McNemar’s test for dependent samples. To judge the magnitude of the effects, we used the standardised mean difference Cohen’s d and the mean square contingency coefficient phi [33]. Effect sizes can be classified as small (d ≥ 0.2; φ ≥ 0.1), medium (d ≥ 0.5; φ ≥ 0.3), or high (d ≥ 0.8; φ ≥ 0.5).

Multiple linear regression analyses were conducted to explore which independent variables (described below) affected WA (dependent variable) at t2.

(I) Sociodemographic variables: sex (male/female), level of education (<10/≥10 years), children (no/yes), and monthly net income per household (<3000 €/≥3000€)
(II) Medical variables: comorbidities (no/yes), chemotherapy (no/yes), and time of sick leave (≤6 months/>6 months since diagnosis)
(III) Psychosocial variables: COPSOQ (0-100) and PACIS (0-100)

All variables (except having children) were selected for inclusion in the regression model as these factors were known to affect work ability in young adults and older cancer populations [9, 12, 13, 34–37]. Having children was included due to its relevance to the AYAs’ age range.

## Results

### Sample characteristics

After screening for the inclusion criteria, N = 762 participants provided written consent. Due to n = 185 dropouts (n = 43 withdrew their consent, n = 88 did not meet the inclusion criteria in a secondary screening, and n = 54 did not complete the questionnaire), the sample size at the time of the initial survey (t1) was N = 577. Between t1 and t2, the drop-out rate was 11% (n = 63). More detailed information on sample recruitment and characteristics is provided by Leuteritz et al [18]. Thus, a total of N = 514 persons participated in the study on both survey dates. In the statistical analysis, only participants with complete values for their WA (WAI item 1) at t1 and t2 were included (N = 502), regardless of their current employment status. Sociodemographic and medical variables are shown in Table 1.

**Table 1:** Baseline and follow-up characteristics of the study sample (N=502)

	N (t1)	%	N (t2)	%
Sociodemographic variables				
Age at diagnosis				
M; SD	29.7	6.1		
Median; range	29.9	18.0 –39.9		
Sex				
Male	127	25.3		
Female	375	74.7		
Highest educational degree				
No school completion/ < 10 years	33	6.6		
≥ 10 years	465	92.6		
Employed or studying	457	91.0	418	83.3
Children (yes)	166	33.1	173	34.5
Monthly net income per household				
< 3000 Euro	292	58.2	287	57.2
≥ 3000 Euro	143	28.5	164	32.7
Recruitment				
Rehabilitation clinics	186	37.1		
Acute clinics	59	11.8		
Tumour registries	154	30.7		
Self-registration (e.g. homepage, e-mail)	103	20.5		
Medical characteristics				
Time since diagnosis in months (M; SD)	12.15	8.21		
Treatment completed	466	92.8		
Cancer diagnosis				
Breast cancer	135	26.9		
Gynaecological cancer	44	8.8		
Testicular cancer	41	8.2		
Thyroid cancer	29	5.8		
Melanoma	17	3.4		
Hodgkin lymphoma	92	18.3		
Non-Hodgkin lymphoma	32	6.4		
Haematological cancer	35	7.0		
Sarcoma	20	4.0		
Gastrointestinal cancer	24	4.8		
Other	33	6.6		
Comorbidities (yes)	99	19.7	83	16.5
Metastases or recurrence (yes)	90	17.9	33	6.5
Therapy				
Chemotherapy^a^	381	75.9		
Radiotherapy^b^	241	48.0		
Surgery	372	74.1		
Time of sick leave since diagnosis				
≤ 6 months			187	37.3
> 6 months			297	59.2

Abbreviations: *M* mean, *SD* standard deviation

^a^Includes radiochemotherapy

^b^Includes radiochemotherapy and nuclear therapy

### Work ability and cognitive impairments

The mean levels of WA and CRCI at t1 and t2 are displayed in Table 2. We found reduced WA (cut-off < 8) in n = 380 (76%) participants at t1 and in n = 287 (57%) at t2. The WA significantly improved from t1 (M = 6.0; SD = 2.3) to t2 (M = 6.8; SD = 2.2; t(501) = −9.3; p < .01). With regard to cognitive functioning, n = 88 (18%) reported CRCI at t1 and n = 82 (16%) at t2.

**Table 2:** Work ability and psychosocial characteristics of the sample at t1 and t2 and differences (N=502)

		T1		T2	t	p	d/φ
	M	SD	M	SD			
WAI	6.0	2.3	6.8	2.2	−9.3	**<. 001**	d = .38
COPSOQ	36.2	22.8	35.2	23.0	1.3	.216	d = .05
PACIS	32.9	30.6	27.7	29.5	3.5	**<. 001**	d = .18
	N	%	N	%	χ^2 a^		
WAI < 8	380	76%	287	57%	56.8	**<. 001**b	φ = .39
COPSOQ > 60	88	18%	82	16%	.3	.594b	φ = .38

Abbreviations: *M* mean, *SD* standard deviation, *t* t-statistic (Student’s t-test), *p* type-I-error probability, *d* effect size Cohen’s d, *φ* effect size phi coefficient, *WAI* Work Ability Index item 1 (scale 0–10), *COPSOQ* mean score cognitive impairments (scale 0–100), *PACIS* Perceived Adjustment to Chronic Illness Scale (scale 0–100) ^a^Frequency differences were tested using McNemar’s test for dependant samples

^b^Continuity corrected

Bold type indicates statistical significance

### Differences in work ability and cognitive impairments for sample subgroups

At t2, the women had lower WA (women: M = 6.7, SD = 2.2; men: M = 7.2, SD = 2.1; F(1, 500) = 5.296, p = .022, η^2^ = .01) than men (Table 3). Furthermore, AYAs with children showed significantly lower WA than childless AYAs at t2 (having children: M = 6.5, SD = 2.5; no children: M = 7.0, SD = 2.1; F(1, 500) = 6.682, p=.010, η^2^ = .01). The AYAs with both physical and psychological work demands had lower WA (M = 6.3; SD = 2.4) than those with mainly physical (M = 7.3; SD = 2.1; p = .004) or mainly psychological work demands (M = 7.2; SD = 1.9; p < .001).

**Table 3:** Univariate associations (ANOVA) of work ability and cognitive impairments of the AYA sample at t2 (N=502)

	Work ability (WAI item 1)					Cognitive impairments (COPSOQ)				
	M	SD	F	p	η^2^	M	SD	F	p	η^2^
Sex										
Men	7.2	2.1	5.3	**.022**	.01	29.3	21.1	11.2	**.001**	.02
Women	6.7	2.2				37.2	23.3			
Age at interview (T2)										
18–27 years	7.1	1.8	2.9	.087	.01	34.9	21.8	0.4	.835	.00
28–43 years	6.7	2.4				35.3	23.6			
Having children										
Yes	6.5	2.5	6.7	**.010**	.01	35.9	23.5	0.3	.598	.00
No	7.0	2.1				34.8	22.8			
Demands of work										
More physical strain	7.3	2.1	10.0	**<. 001**a	.04	31.3	24.7	0.9	.393	.00
More psychological strain	7.2	1.9				35.9	22.6			
Physical and psychological	6.3	2.4				35.1	23.0			
Tumour										
Solid	6.7	2.3	1.6	.211	.00	35.8	23.7	0.7	.392	.00
Non-solid	7.0	2.1				33.9	21.5			
Chemotherapy										
Yes	6.7	2.3	4.5	**.035**	.00	35.8	22.6	1.3	.262	.00
No	7.2	2.0				33.2	24.1			

Abbreviations: *M* mean, *SD* standard deviation, *F* F-statistic (ANOVA), *t* t-statistic (Student’s t-test), *p* type-I-error probability, *η*^*2*^ effect size partial eta squared, *WAI* Work Ability Index (scale 0–10), *COPSOQ* mean score cognitive impairments (scale 0–100). Bold type indicates statistical significance

^a^Post hoc tests with Bonferroni correction showed significant differences between more physical (p = .004) and psychological strain (p < .001) compared to equally physical and psychological strain

### Predictors for work ability at t2

Lower WA at t2 was associated with sociodemographic (having children), medical (having comorbidities, time of sick leave > 6 months since diagnosis), and psychosocial variables (higher CRCI and higher effort coping with the disease). The variable CRCI showed the largest standardised regression coefficient (β = −.34; p < .01). As much as 48% of the variance in WA at t2 could be explained by the regression model (adjusted R^2^ = .48). The model’s prediction ability was statistically significant with F(9, 407) = 42.79, p < .01 (Table 4).

**Table 4:** Results of multiple linear regression analysis for work ability (WAI item 1) at t2 (N = 502)

adj. R^2^ = 0.48	Beta β	p-value
Sociodemographic		
Sex^1^	−.03	.499
Educational degree^2^	.06	.120
Children^3^	−**.08**	**.036**
Monthly net income per household^4^	.06	.137
Medical		
Comorbidities^5^	−**.20**	**<. 001**
Chemotherapy^6^	−.01	.757
Time of sick leave^7^	−**.12**	**.002**
Psychosocial		
Cognitive impairments (COPSOQ)^8^	−**.34**	**<. 001**
Effort coping with the disease (PACIS)^9^	−**.28**	**<. 001**

Abbreviations: *β* standardised beta coefficient, *p* type-I-error probability, reference categories

^1^Female sex

^2^≥ 10 years

^3^Yes

^4^≥3 000 €

^5,6^Yes

^7^>6 months

^8,9^0–100

Bold type indicates statistical significance

## Discussion

The aim of this study was to investigate AYAs’ WA and CRCI as well as their development during the course of cancer. In addition, the investigation sought to identify the risk factors associated with reduced WA in the long term.

### Work ability

The study showed that subjectively perceived WA at both measurement points was reduced and a substantial proportion of the AYAs (T1: 76%; T2: 57%) required intervention. The WA of the overall sample improved over 1 year. This corresponds to previous studies that identified reduced WA after completing treatment for both younger and older cancer patients [9, 12, 36]. In the work of Dahl et al. [12], AYAs even reported limited WA values 16 years (2019) after their diagnosis. This indicates that AYAs may not achieve maximum WA even after the period selected for our study. The negative effects of cancer on WA are most pronounced during treatment and immediately afterwards, and then they had decreased 1 year later, but the WA remains persistently. This effect on WA may be due to numerous disease and treatment-related long-term effects (e.g. radiation injuries, pain, polyneuropathy, depression, and anxiety) [12]. In addition, cancer-related fatigue is a common and persistent symptom among AYAs [38]. One recent study on AYAs found that an increased number of long-term effects and an increased level of depression and fatigue were negatively associated with WA [12]. This underlines the long-term care and intervention needs of AYA cancer survivors with regard to their WA.

### Cognitive impairments

At both measurement points, AYA cancer survivors reported that, on average, they rarely had to deal with CRCI. Less than one-fifth of the respondents reported frequent or permanent CRCI during the last 4 weeks. This means that cognitive difficulties are relevant in the long term for every fifth patient. In another study, more AYAs reported cognitive problems [14]. This can be attributed to the following discrepancies. Not only do the measuring instruments for assessing CRCI differ, but we also asked about difficulties in decision-making and thinking clearly. In addition, the samples differ in sex ratio and time since diagnosis. However, when looking at subgroups, we found that women were more affected by CRCI than men, which could be attributed to neurocognitive differences. Ruigrok et al. [39] explained differences in cognition according to gender by referring to differences in brain structure in healthy men and women, which may affect the cognitive effects of cancer and its treatment. However, the interpretation of this subgroup difference is limited. The mean extent of CRCI in the present sample remained stable over the course of 1 year. This is a common observation. Up to 53% of AYA cancer survivors report treatment-related cognitive dysfunction, which may persist for up to 25 years post-diagnosis [7, 14, 40]. This seems to be caused by unique neurological processes in young adulthood. The brain, particularly the frontal lobe that is significantly involved in cognitive functions, is not completely mature until the early 20s. Therefore, cancer treatment at this age may have a negative and long-lasting impact on neurological development [16]. There is also evidence that some cytostatics can cause long-lasting neurologic deficits (e.g. methotrexate [41]).

### Associations

Psychosocial factors were the most closely associated with the long-term extent of WA, even more so than medical factors. The presence of CRCI turned out to be the strongest predictor of long-term reduced WA in the AYAs. This result is consistent with the findings of quantitative studies on older adults [35] and qualitative studies on AYAs [9]. Therefore, screening the cognitive functional levels of AYAs in the context of aftercare is imperative, as concentration problems and memory and attention disorders can restrict WA for months, if not years, after treatment [35]. In this way, CRCI could be countered in the sense of reduction or even compensation. Bains and colleagues [37] reported that experiencing self-efficacy and competence in relation to certain tasks or functions has positive effects on WA. In addition to cognitive training, the targeted promotion of this cognitive-psychological construct could prove to be a useful clinical concept.

The study also found that participants who exerted higher effort to cope with the disease reported increased limitations to their WA. This seems understandable in view of the fact that mental stress is one of the most common causes of work incapacity [42]. The process of coping with cancer—even beyond treatment—is therefore of great importance to improve WA to a level that meets the specific requirements of the workplace. The AYAs need more help on this issue, on the one hand, because of the higher psychological burden of cancer compared with older people [43] and, on the other hand, because AYAs are not facing (age-related) early retirement, as is the case in older patients.

Although the negative impact of comorbidities on the WA of older adults is commonplace [34], this study was able to confirm a correlation with AYAs. This underlines the clinical relevance of support to manage symptoms in AYA cancer survivors, not only during therapy but also during the remission phase [12].

Young affected mothers and fathers seemed to feel less able to cope with professional demands than childless AYAs. This supports the theoretical assumption that the upbringing and care of (in the case of AYAs) generally young children requires additional resources from the AYAs, which are ultimately reduced in the workplace. This finding implies the need to offer targeted, and possibly even family-centred, support and care, especially to young cancer patients who are already parents, which can contribute to the long-term strengthening of their resources and capacities in the professional field.

### Limitations

Although this study was done on a large sample, which was comparable in terms of its distribution of age and cancer sites with the nationwide German AYA population [18], the results should be interpreted against the background of the following limitations. First, women and a high level of education were overrepresented in the study. It seems to be a common phenomenon that men are less likely to participate in clinical trials [44]. We discussed the different participation rates earlier [18]. In addition, the option for patients to register themselves for the study might have resulted in a selection bias, because it is possible that the topic of the study had greater relevance to patients who have more limitations in the investigated fields. Also, the COPSOQ has not been validated in cancer populations; therefore, we recommend appropriate validation studies among cancer patients and survivors. Moreover, the data on current WA compared to the ‘maximum ever achieved’ might have been prone to recall bias or socially desirable responses. Finally, it cannot be ruled out that the significant effect of the children was age related. In the subgroup analysis, age showed no clear effect, but, because of the unbalanced distribution of own children across the age groups (more children among older participants), no definite conclusions could be drawn. Moreover, it is conceivable that marital status could also be related to this effect.

### Implications for research and practice

Future research should explore the role of other factors, such as employer support or additional work-related and psychosocial factors, that might have a long-term negative impact on the WA of AYAs and examine what support AYAs need to successfully re-enter education or employment. Comparisons with healthy peers would also be helpful. Health care providers and psychosocial health services should address possible reduced WA in AYA cancer survivors (especially in risk patients), even 2 years (on average) post-diagnosis and after returning to work. The application of specific intervention programmes for tumour-associated CRCI represents an important research topic for the future. There is a need for cancer survivorship concepts, designed for the longer term and able to be used more flexibly by AYA cancer survivors, depending on the time of return to work or changes in the job. In addition, employers have to be educated to identify and better respond to the psychosocial needs of AYA cancer survivors at work. Therefore, multi-professional cooperation is desirable and could provide significant support to AYA cancer survivors in professional reintegration.

## Conclusion

In summary, the results support the assumption that cancer is a life-changing experience with lasting consequences, also for the professional futures of many AYA cancer survivors. Achieving maximum WA is a major challenge for those affected, even after completing treatment. Psychosocial support for AYA cancer survivors in professional reintegration and regaining WA is therefore essential and represents a field of research that deserves further attention in the future. In particular, cognitive skills and disease management should be promoted in the context of a multi-professional cancer survivorship concept for AYAs.

## Supplementary Information

ESM 1 (DOCX 26 kb)

## Data Availability

The dataset, which the study is based on, is publicly not available due to required data protection but is available upon reasonable request with a signature of a data privacy form. To request the data, the reader may contact the first author.
